# Emergence of NDM-1 on IncR-IncFIA (HI1) plasmid in carbapenem-resistant *Kluyvera ascorbata* from China

**DOI:** 10.3389/fmicb.2025.1666215

**Published:** 2025-09-11

**Authors:** Duo Zhang, Di Zhou, Shuai Zheng, Xindan Zhang, Qinlong Hou, Yang Liu, Shuang Li, Huiming Han

**Affiliations:** ^1^The School of Basic Medicine, Beihua University, Jilin, China; ^2^The Center for Infection and Immunity, Beihua University, Jilin, China; ^3^Department of Pathophysiology, School of Basic Medical Sciences, Beihua University, Jilin, China; ^4^Department of Science and Education, Affiliated Hospital of Beihua University, Jilin, China

**Keywords:** *Kluyvera ascorbata*, NDM-1, CTX-M-77, IncR, IncFIA(HI1)

## Abstract

New Delhi metallo-β-lactamase 1 (NDM-1), a member of the B-type metallo-β-lactamase (MBL) family, emerged as a major focus in resistance research, raising serious concerns about the treatment of bacterial infections over the past decade. *Kluyvera ascorbata*, generally considered a bacterium of low pathogenicity and rarely associated with severe infections, has nonetheless demonstrated significant resistance to a broad spectrum of antibiotics in recent studies. In this study, we successfully isolated a *K. ascorbata* strain harboring the antibiotic-resistant genes *bla*_NDM-1_ and *bla*_CTX-M-77_ from a fecal sample of a patient with diarrhea in China. The strain was accurately identified using matrix-assisted laser desorption/ionization time-of-flight mass spectrometry (MALDI-TOF MS). Additionally, the minimum inhibitory concentration (MIC) of the strain against various antimicrobial agents was determined using agar dilution and microdilution method. The results indicated that the strain exhibited resistance to all tested antimicrobial agents. The *bla*_NDM-1_ resistance gene was located on an IncFIA (HI1), IncR plasmid, as revealed by whole-genome sequencing. A detailed analysis of the plasmid’s size, number, and location was conducted using S1-nuclease pulsed-field gel electrophoresis (S1-PFGE), Southern blotting, and conjugation experiments. These experiments successfully demonstrated the transfer of the plasmid carrying *bla*_NDM-1_ into the recipient bacterium *Escherichia coli EC600.* These findings underscore the urgent need for continuous surveillance of the *bla*_NDM-1_-carrying plasmid in clinical isolate of *K. ascorbata* to prevent and contain its further dissemination in China.

## 1 Introduction

*Kluyvera ascorbata*, a Gram-negative bacillus, has been increasingly recognized in recent years as an opportunistic pathogen of potential clinical relevance, particularly among immunocompromised individuals. The genus Kluyvera was initially identified as a novel taxonomic group corresponding to the previously termed “Enteric Group 8” (also known as API Group 1) (Farmer et al., 1981). Similar to most members of the family Enterobacteriaceae, Kluyvera species exhibit typical phenotypic characteristics: they are Gram-negative, motile via peritrichous flagella, catalase-positive, oxidase-negative, capable of growing on MacConkey agar, and ferment d-glucose with the production of acid and gas. These organisms were historically considered susceptible to a wide range of antibiotics (Ulloa-Clavijo et al., 2023; Ochi et al., 2017; Lee et al., 2019; Akiki et al., 2023). In recent years, with the development of molecular phylogenetics, researchers have employed methods such as 16S rRNA sequencing, multilocus sequence analysis (MLSA), and whole-genome sequencing (WGS) to further elucidate the phylogenetic relationship between the genus Kluyvera and Enterobacter. These studies have shown that the two genera share a close evolutionary relationship on the phylogenetic tree. Many strains that were historically classified as Enterobacter have since been reassigned to *Kluyvera*. This taxonomic ambiguity not only increases the difficulty of clinical identification but may also lead to an underestimation of the epidemiological significance of *Kluyvera* species (Farmer et al., 1981; Pavan et al., 2005; Brady et al., 2013). However, due to the high degree of phenotypic similarity with other members of the *Enterobacteriaceae*, accurate identification of *Kluyvera* species in clinical microbiology laboratories remains a diagnostic challenge (Bellaoui et al., 2009).

Although *Kluyvera* spp. are infrequently isolated in medical microbiology and human infections have historically been considered rare with unclear clinical significance, increasing reports indicate that these organisms not only possess opportunistic pathogenic potential but may also be involved in the dissemination of antibiotic resistance genes (Altun Koroglu et al., 2010; Stock, 2005). Antimicrobial resistance in *K. ascorbata* has become a major challenge in antimicrobial therapy. Numerous studies have demonstrated that *K. ascorbata* has developed resistance to multiple classes of antibiotics, including commonly used classes such as beta-lactams, carbapenems, aminoglycosides, fluoroquinolones, and tetracyclines (Rodríguez et al., 2004; Rodriguez et al., 2018; Mutoh et al., 2019).

Notably, NDM-1 (New Delhi metallo-β-lactamase-1) is an enzyme capable of degrading various antibiotics, particularly carbapenems. This enables strains carrying the *bla*_NDM-1_ gene to exhibit significant multidrug resistance (Gao et al., 2023; Marquez-Ortiz et al., 2017). The rapid and widespread dissemination of NDM-1 has positioned it as a critical concern in the global antibiotic resistance crisis (Guo et al., 2011; Heng et al., 2024). This phenomenon presents considerable challenges to clinical treatment and poses substantial threats to public health and safety (Adams et al., 2020; Hirabayashi et al., 2021; Liu et al., 2023). Although *K. ascorbata* is not considered a highly pathogenic bacterium in the traditional sense, it may serve as a reservoir of antibiotic resistance genes in the environment or intestinal microbiota, contributing to the interspecies dissemination of resistance determinants such as *bla*_NDM-1_. *Kluyvera* species often harbor mobile genetic elements, including plasmids, integrons, and transposons, which enable the horizontal transfer of resistance genes to other *Enterobacteriaceae* pathogens, such as *Escherichia coli* and *Klebsiella pneumoniae*. Therefore, its potential “silent role” in the dissemination network of NDM-1 resistance should not be overlooked (Raro et al., 2019; Li et al., 2019).

This study focused on *K. ascorbata* L4110hy, the first *K. ascorbata* strain identified to carry the *bla*_NDM-1_ gene in China. The present research not only elucidates the drug resistance background of *K. ascorbata* in clinical isolates and clarifies its resistance mechanisms, which are crucial for effective clinical treatment and infection control. Therefore, it is essential to implement a continuous monitoring program for the prevalence and spread of *bla*_NDM-1_ and other drug-resistant genes conferring carbapenemase-like activity in *K. ascorbata* and other bacterial populations to prevent potential public health crises.

## 2 Materials and methods

### 2.1 Sample collection and bacterial culture

In 2021, a carbapenem-resistant strain of *K. ascorbata* (strain L4110hy) was isolated from a stool sample of a male patient with severe diarrhea at a teaching hospital in Zhejiang Province. The strain was incubated on MacConkey agar plates at 37°C for 18 to 24 h. Subsequently, individual colonies were screened using matrix-assisted laser desorption ionization time-of-flight mass spectrometry (MALDI-TOF) and polymerase chain reaction (PCR) was performed to detect the carbapenemase gene *bla*_NDM-1_ in the isolates.

### 2.2 Location of the *bla*_NDM-1_ gene and transferability of plasmid carrying *bla*_NDM-1_

The number and size of plasmids in strain L4110hy were determined using the S1 nuclease pulsed-field gel electrophoresis (S1-PFGE) method. The location of the *bla*_NDM-1_ resistance gene was assessed by Southern blotting and hybridization with an NDM-1-specific probe, using Salmonella H9812 as a reference (Ge et al., 2023). In the conjugation experiments, rifampicin-resistant *Pseudomonas aeruginosa PAO*, *Pseudomonas aeruginosa Ri,* and *Escherichia coli EC600* were used as recipients, and the plasmid transferability using the broth method (Chen et al., 2024; Liu et al., 2025). The recipient bacteria and target single colony were inoculated into 5 mL LB broth and cultured at 37°C with shaking for approximately 6 h. The donor and recipient bacteria were evenly mixed in a 2:1 ratio in 5 mL LB broth and incubated at 37°C for approximately 18 h. Transconjugants were selected on Mueller-Hinton medium containing [400 mg/L rifampicin and 2 mg/L meropenem] and [2 mg/L meropenem, and 2 mg/L mucin]incubated at 37°C for 18 h. Successful transfer was verified by detecting the *bla*_NDM-1_ target gene using polymerase chain reaction (PCR). Additionally, strain identification was performed using matrix-assisted laser desorption/ionization time-of-flight mass spectrometry (MALDI-TOF/MS), and drug sensitivity assays were conducted to confirm the successful transfer of the plasmids. The transfer efficiency was calculated by dividing the number of transconjugants (CFU/mL) by the number of donor bacteria (CFU/mL) (Zhang et al., 2023).

### 2.3 Drug sensitivity test

The minimum inhibitory concentration (MIC) of the fixed strain L4110hy against a range of antimicrobial drugs was determined using two complementary methods: agar dilution and microdilution method. The results of the drug sensitivity experiments were interpreted in accordance with the guidelines established by the Clinical and Laboratory Standards Institute (CLSI) and the European Committee for Antimicrobial Susceptibility Testing (EUCAST)^1^. The interpretation of tigecycline, omadacycline, and eravacycline was based on definitions provided by the FDA (Tigecycline Injectable Products, Omadacycline Injectable and Oral Products, and Eravacycline Injectable Products). *E. coli* ATCC25922 was used as a quality control strain.

### 2.4 Whole-genome sequencing and bioinformatics analysis

To obtain the complete genome sequence of the strain, we initially extracted the genomic DNA using the QIAGEN Bacterial DNA kit. Subsequently, DNA sequencing was performed using both the Illumina NovaSeq 6,000 platform and Oxford Nanopore technology. The sequenced fragments were assembled using Unicycler v0.4.7 software^2^, and the assembled genome was annotated using the RAST 2.0 online tool^3^.

To gain a deeper understanding of the genetic characteristics of the strain, we identified insertion elements (IS) using the ISfinder^4^ database and detected acquired antibiotic resistance genes (ARGs) and plasmid incompatibility types through the ResFinder^5^ and PlasmidFinder^6^ databases (Carattoli et al., 2014; Zankari et al., 2012).

The oriTfinder tool was employed to investigate the origin of transfer (oriT^7^) within the DNA sequences of bacterial mobile genetic elements. Concurrently, the VFDB.1^8^ database was used to identify virulence factors. For further insight into the plasmid sequences, we compared them with the GenBank database using the BLASTN tool^9^ (Liu et al., 2024).

The BLAST Ring Image Generator (BRIG) and EasyFig software were utilized to construct the circular map for plasmid comparison and to perform comparative mapping of the genetic environment surrounding the *bla*_NDM-1_ gene, respectively (Liu et al., 2024; Luo et al., 2024; Liu et al., 2024; Chen et al., 2024). Based on the blast analysis results of pL4110, 72 plasmid sequences were downloaded from the NCBI database. All sequences were annotated using Prokka, and a phylogenetic tree was constructed using the maximum likelihood method in MEGA 11 software.

## 3 Results

### 3.1 Isolation and characterization of L4110hy carrying *bla*_NDM-1_

In 2021, a male patient was admitted to a teaching hospital in Zhejiang Province for the treatment of severe diarrhea and vomiting. During his treatment, a bacterial strain designated L4110hy was isolated from the patient’s fecal sample. Mass spectrometry identified the strain as *K. ascorbata*, which demonstrated resistance to carbapenems, as documented in clinical reports. Subsequent PCR testing confirmed the presence of the *bla*_NDM-1_ gene, marking the first instance of NDM-1-producing *K. ascorbata* identified in China.

### 3.2 Drug sensitivity results of L4110hy

L4110hy and EC600-pL4110-NDM-1 exhibited decreased susceptibility to imipenem, meropenem, ceftriaxone, cefotaxime, ceftazidime, and piperacillin-tazobactam; however, these strains remained susceptible to aztreonam. In contrast, all strains showed good sensitivity to amikacin, gentamicin, chloramphenicol, and trimethoprim-sulfamethoxazole. Notably, the EC600 strain demonstrated significantly higher sensitivity to fluoroquinolones (such as ciprofloxacin and levofloxacin) and chloramphenicol, with greater sensitivity compared to both L4110hy and EC600-pL4110-NDM-1. Their antimicrobial susceptibility testing results are shown in Table 1.

**Table 1:** Results of drug sensitization experiments.

Antibiotics	MIC value (μg/mL)		
	L4110hy	EC600-pL4110-NDM-1	EC600
Aztreonam	0.5	0.5	0.06
Imipenem	4	4	0.125
Meropenem	4	4	0.06
Ceftriaxone sodium	64	128	0.125
Cefotaxime sodium	128	128	0.125
Ceftazidime	128	128	0.5
Levofloxacin	0.25	0.25	0.06
Ciprofloxacin	0.25	0.25	0.008
Amikacin	4	2	1
Gentamicin	1	1	1
Piperacillin Sodium and Tazobactam Sodium	128	128	4
Fosfomycin + Glucose 6-phosphate	1	1	1
Chloramphenicol	32	32	8
Trimethoprim-sulfamethoxazole	≤0.125	≤0.125	≤0.125
Cefepime	8	8	0.06
Ceftazidime/Avibactam	>128	>128	0.25
Tigecycline	0.5	0.5	0.125

### 3.3 Genomic characterization of L4110hy

Table 2 provides a summary of the genomic characterization of L4110hy, which contains a circular chromosome of 4,845,762 bp with an average GC content of 54%. Additionally, the genome comprises six plasmid sequences, with lengths ranging from 1,554 bp to 301,733 bp. The resistance genes carried by the strain were identified through the analysis of antibiotic resistance genes (ARGs) using the ResFinder tool. The chromosome of L4110hy carries the *bla*_CTX-M-77_ resistance gene, while the plasmid carries the *bla*_NDM-1_ resistance gene. The chromosome harbors the virulence factor *nlpI*, while the plasmid carries the virulence factors *clpK*1 and *terC*.

**Table 2:** Genomic characterization of L4110hy.

Contig	Total number of bases (bp)	G + C content (%)	Plasmid replicon type	Transfer region	Resistance genes	Virulence factors
*K. ascorbata* strain L4110hy 1	4,845,762	54	NA	/	*bla*_*CTX-M-7*7_	*nlpI*
*K. ascorbata* strain L4110hy 2	301,733	48	Col440I, IncFIB(K), IncHIIA, IncHIIB(R27)	IncHII1B(R27)	/	*clpK1*, *terC*
*K. ascorbata* strain L4110hy 3	52,121	52	IncFIA(HI1), IncR	/	*bla*_NDM-1_	/
*K. ascorbata* strain L4110hy 4	4,019	46	NA	/	/	/
*K. ascorbata* strain L4110hy 5	2,723	46	Col440I	/	/	/
*K. ascorbata* strain L4110hy 6	2,206	46	NA	/	/	/
*K. ascorbata* strain L4110hy 7	1,554	47	NA	/	/	/

### 3.4 Characterization of plasmids containing *bla*_NDM-1_

**Figure 1:**
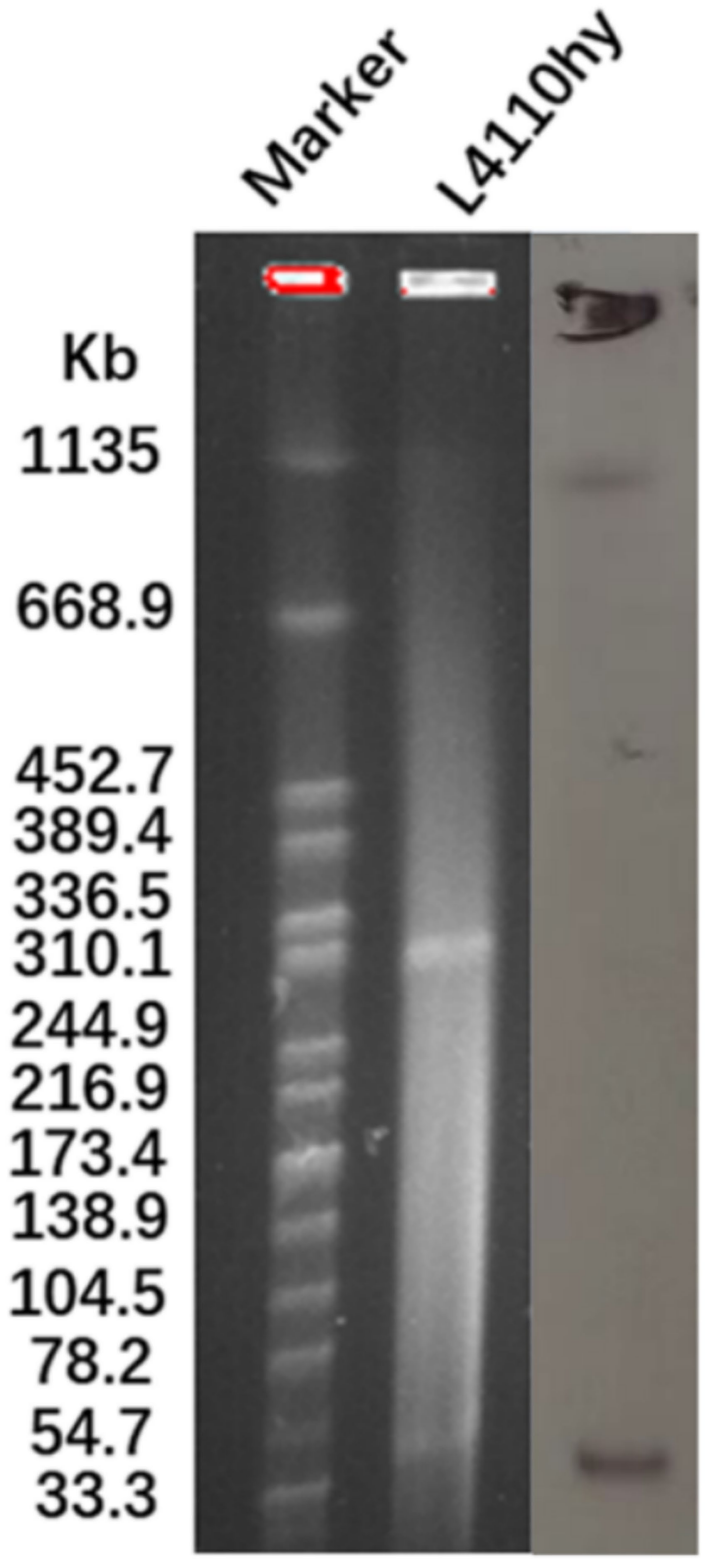
The plasmid size of L4110hy was determined using S1-PFGE with *Salmonella enterica* serotype Braenderup H9812 as a size marker. Subsequently, Southern blot hybridization was performed using an NDM-1-specific probe.

**Figure 2:**
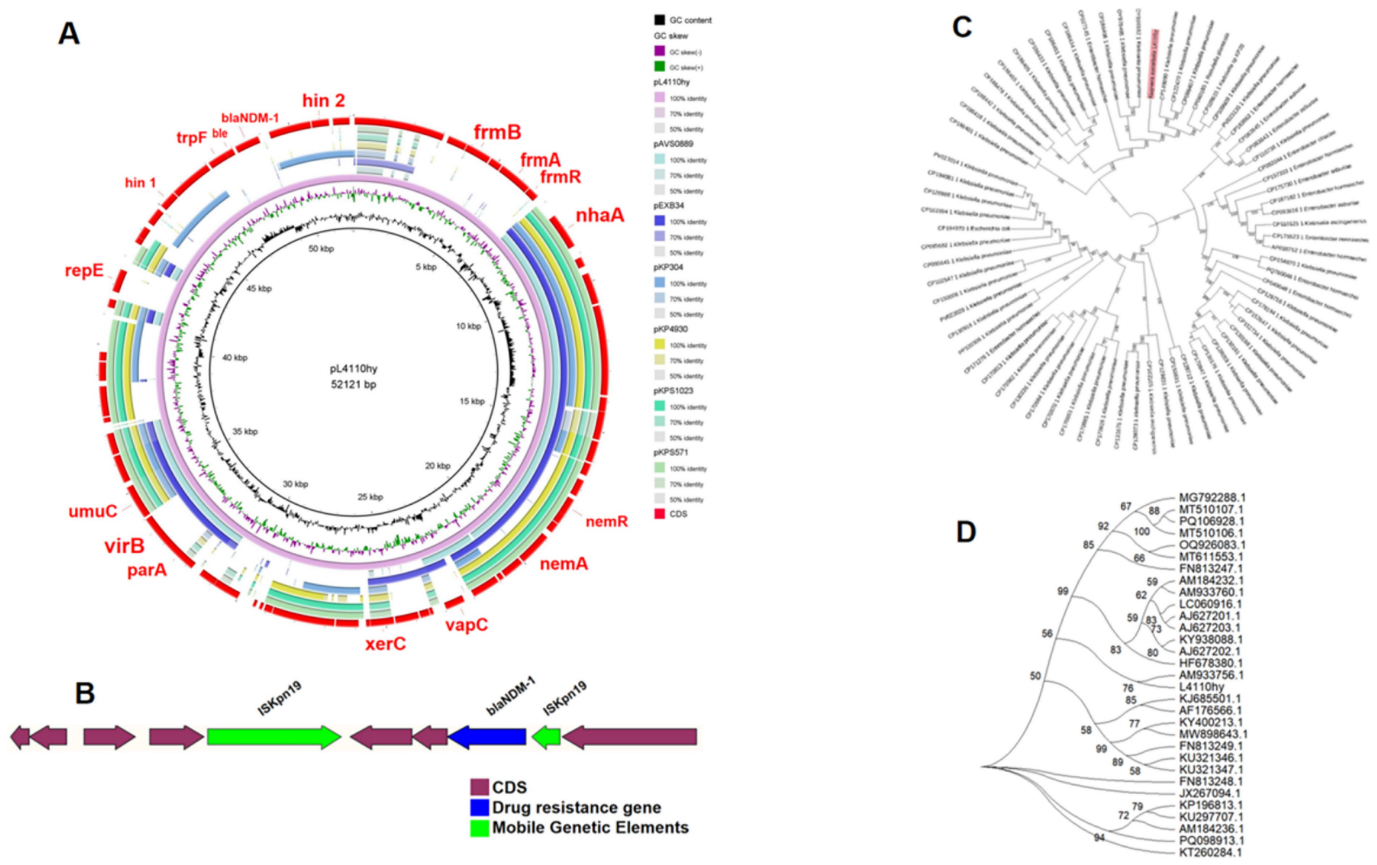
**(A)** Circular comparative alignment of plasmid pL4110-NDM-1 with four reference plasmids from *K. ascorbata* using BRIG. Although the nucleotide identity of these plasmids in the blaNDM-containing region exceeded 99% compared with pL4110-NDM-1, the overall query coverage was only over 45%, indicating the existence of structural diversity beyond the resistance modules. **(B)** Genetic context of *bla*_NDM-1_ showing IS*Kpn19* elements flanking the resistance gene, suggestive of transposon-mediated mobility. **(C)** Through BLAST analysis, 72 plasmid sequences similar to pL4110-NDM-1 were screened. A phylogenetic analysis of L4110hy was performed using the maximum likelihood method, and the phylogenetic analysis and visualization were carried out on MEGA11. **(D)** Phylogenetic relationship between strain L4110hy and other *K. ascorbata* strains.

The *bla*_NDM-1_ resistance gene was located on a plasmid with a total length of 52,121 bp (Figure 1), as determined by both ResFinder and S1-PFGE analyses. This plasmid was classified as an IncR-IncFIA composite plasmid. BLASTN searches were performed using the plasmid pL4410-NDM-1, which contains the *bla*_NDM-1_ resistance gene, as a reference sequence in the NCBI database. By searching against the core plasmid region in GenBank, the pL4110-NDM-1 backbone and *Enterobacter hormaechei* strains pEXB34 (PQ760046.1), pAVS0889 (CP092044.1), *Klebsiella pneumoniae* strains pKPS1023 (CP186455.1), pKPS571 (CP186467.1), pKP4930 (CP186394.1), pKP304 (CP178194.1) showed very high nucleotide identity (>99%), Moreover, their Query Cover exceeds 45%. Genetic environment analysis showed that IS*Kpn19* was located upstream and downstream of the drug resistance gene *bla_NDM-1_* (Figures 2A,B). The phylogenetic tree constructed based on the core genome in this study reveals the evolutionary position of the host strains carrying the pL4110-NDM-1 plasmid and its genetic association with closely related species. This plasmid forms an independent branch in the phylogenetic tree and is critically clustered closely with a group of *K. pneumoniae* plasmids, suggesting that the L4110hy strain is substantially phylogenetically close to the *K. pneumoniae* branch. Combining the clustering results, it can be speculated that the origin or early evolutionary process of pL4110-NDM-1 is significantly associated with a specific plasmid pool of *Klebsiella pneumoniae* (Figure 2C). To analyze the phylogenetic relationship between strain L4110hy and other *K. ascorbata* strain, we used the 16S sequence of strain L4110hy as a template for BLAST alignment in the NCBI database. The analysis results are shown in Figure 2D. Strain L4110hy is closely related to AM933756.1. Strain AM933756.1 is from Argentina. Through genomic analysis, we found that it does not carry the *bla*_NDM-1_ resistance gene. Strain L4110hy is closely related to it, and the uneven distribution of the *bla*_NDM-1_ gene strongly suggests that this resistance gene is not an inherent part of the genetic background of the *K. ascorbata* species, but was recently acquired through a horizontal gene transfer mechanism. After analyzing the resistance genes of *K. pneumoniae* CP149890.1, CP122470.1, CP099407.1, CP109609.1, CP109615.1, and *Raoultella planticola* CP040185.1, it was found that these plasmids do not carry *bla_NDM-1_* (Supplementary Table 1).

**Figure 3:**
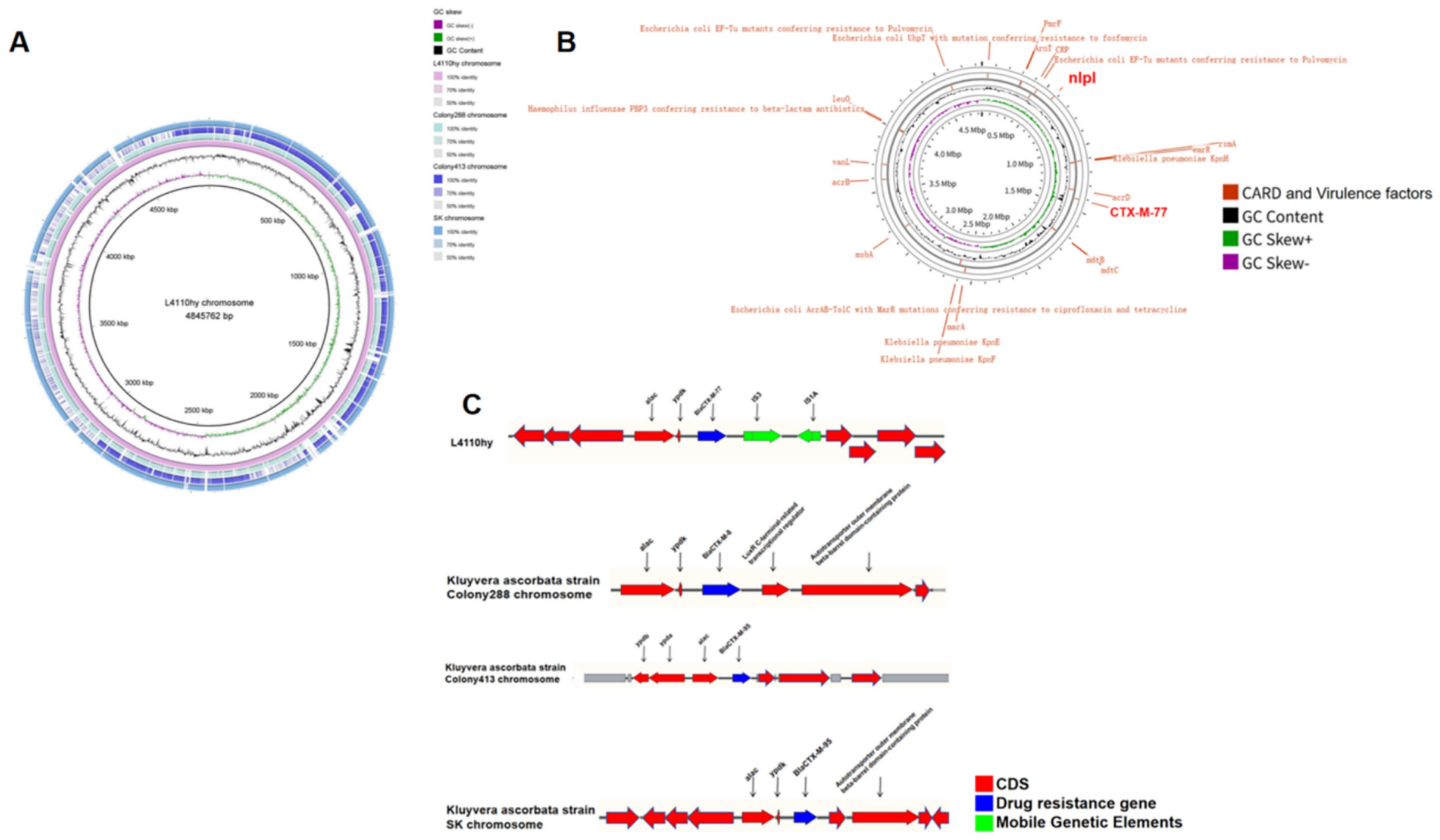
Chromosomal structure and comparative analysis of L4110hy. **(A,B)** Genome-wide circular alignment of the L4110hy chromosome with three *K. ascorbata* reference strains. The alignment highlights conserved regions (marked in blue) and divergent regions (marked in red), with a focus on the core genomic structure and the genetic loci potentially involved in horizontal gene transfer. Notably, the area surrounding *bla*_CTX-M-77_ is marked to show the genomic conservation across strains. **(C)** Genetic context of the *bla*_CTX-M-77_ resistance gene on the L4110hy chromosome. The resistance gene is located between the insertion sequences IS*3* and IS*1A*, which are known to mediate horizontal gene transfer. The locations of these insertion sequences are indicated with arrows pointing to their respective positions upstream and downstream of *bla*_CTX-M-7*7*_.

The *bla*_CTX-M-77_ is located on chromosome 4,845,762 bp in length, and a BLASTN search was performed using the L4110hy chromosome of the chromosome containing the *bla*_CTX-M-77_ in the NCBI database as the reference sequence. A BLASTN search was performed using the core sequence region encompassing *bla*_CTX-M-77_ and its flanking genetic context from the L4110hy chromosome as the query sequence in the GenBank database. The results showed that the L4110hy chromosome shared very high query coverage and nucleotide identity (all above 95%) with the chromosomes of *K. ascorbata* strain Colony288 (CP078559.1), strain Colony413 (CP070603.1), and strain SK (CP096201.1). Environmental analyses indicated the presence of IS*3* and IS*1A* insertion sequences downstream of the *bla*_CTX-M-77_ resistance gene (Figure 3). As shown in Figures 3A–C, comparative circular alignment of the L4110hy chromosome with three reference strains (*K. ascorbata* Colony288, Colony413, and SK) revealed strong structural conservation in the chromosomal region harboring *bla*_CTX-M-77_, with both query coverage and nucleotide identity exceeding 95%. Despite this high conservation, detailed genetic context analysis (Figure 3C) identified two well-characterized insertion sequences, IS*3* and IS*1A*, located downstream of *bla*_CTX-M-77_, oriented in tandem. These mobile genetic elements are known mediators of non-homologous recombination and can facilitate horizontal gene transfer and genomic rearrangement. The presence of IS*3* and IS*1A* flanking the *bla*_CTX-M-77_ gene suggests that this resistance determinant may have been mobilized from an extrachromosomal element, such as a plasmid or integrative transposon, and stably integrated into the *K. ascorbata* chromosome. Moreover, such chromosomal integration could confer enhanced genetic stability compared to plasmid-borne resistance genes, especially under non-selective environmental conditions.

### 3.5 Bacterial coupling assay

The EC600-pL4110-NDM-1 coupler was selected for identification and confirmed as *E. coli* using MALDI-TOF mass spectrometry. Subsequently, polymerase chain reaction (PCR) was performed to detect the *bla*_NDM-1_ resistance gene, yielding a positive result. This confirmed that the plasmid carrying the *bla_NDM-1_* resistance gene from the donor strain L4110hy had been successfully transferred to the recipient strain *E. coli* EC600 (the successful transfer experiment of the EC600 recipient strain was conducted using selective antibiotics: 400 mg/L rifampicin and 2 mg/L meropenem), with a conjugation efficiency of 3.87 × 10^−4^. Meanwhile, the transfer experiments with *P. aeruginosa PAO1* and *Ri* as recipient strains were unsuccessful.

## 4 Discussion

This study reports for the first time the discovery of *bla*_NDM-1_ in *K. ascorbata*, a finding that marks the first recorded case in China. This result significantly expands the known host range of *bla*_NDM-1_, emphasizing the potential role of this less-studied species of the Enterobacteriaceae family as a hidden reservoir of carbapenem resistance. As a metal-β-lactamase, *bla*_NDM-1_ can severely impair the efficacy of carbapenem antibiotics, making it one of the major driving factors of the global antibiotic resistance crisis (Jia et al., 2025). Particularly in China, with the increasing prevalence of multidrug-resistant strains, *K. ascorbata* serves as an emerging carrier of resistance genes, posing a potential threat to public health. Our study highlights the need for monitoring non-traditional pathogens, especially in high-selective-pressure environments like hospitals, to effectively prevent the further spread of antibiotic resistance genes.

Genome analysis revealed that *bla*_NDM-1_ is carried on a unique IncR-IncFIA (H11) chimeric plasmid. IncR plasmids are known for their efficient transferability and frequent association with multidrug resistance, while IncFIA plasmids ensure stable replication in the Enterobacteriaceae family, helping maintain the persistence of resistance traits (Zhang et al., 2025; Chatzidimitriou et al., 2025). The combination of these two replicons likely enhances the horizontal transmission and long-term stability of *bla*_NDM-1_. Although the resistance gene locus is highly conserved compared to reference plasmids, the overall structure of the pL4110-NDM-1 plasmid differs, reflecting recombination and gene acquisition events. This showcases the structural plasticity and evolutionary adaptability of plasmids carrying *bla*_NDM-1_.

The gene environment of *bla*_NDM-1_ in *K. ascorbata* differs significantly from that in other known hosts such as *K. pneumoniae* and *E. coli*. Typically, *bla*_NDM-1_ is located within the Tn*125* transposon element, which is flanked by IS*Aba125* insertion sequences, with *trpF* and *dsbD* genes often located downstream (Zhang et al., 2024). These structures facilitate stable transmission and easy transfer, particularly in plasmid-mediated spread. However, in *K. ascorbata*, the surrounding genetic environment of *bla*_NDM-1_ lacks these classic features, notably the absence of the *trpF* and *dsbD* genes downstream. This absence suggests that *bla*_NDM-1_ may have been mobilized by a different mechanism. This observation is closely associated with the presence of the IS*Kpn19* insertion sequence, indicating that IS*Kpn19* may play a key role in the mobilization of *bla*_NDM-*1*_.

IS*Kpn19*, a typical member of the IS*3* family, promotes the horizontal spread of adjacent genes through a “cut-and-paste” or replicative transposition mechanism (Zi et al., 2024). In *K. ascorbata*, IS*Kpn19* is not only located near *bla*_NDM-1_ but may also facilitate the cross-plasmid transfer of *bla*_NDM-1_. Compared to the IS*Aba125* insertion sequence in Tn*125*, the mechanism of IS*Kpn19* is more flexible, promoting recombination and spread of resistance genes through various insertion and transposition events. Specifically, IS*Kpn19’s* insertion sequence can guide the cross-plasmid transfer of *bla*_NDM-1_, potentially even integrating *bla*_NDM-1_ into the chromosome, thus accelerating its horizontal spread among bacterial host (Wang et al., 2025).

This phenomenon is not commonly seen in other known *bla*_NDM-1_ carriers such as *K. pneumoniae* or *E. coli*, which typically rely on the stability of the Tn*125* element to maintain long-term transmission of resistance traits (Marquez-Ortiz et al., 2017; Yao et al., 2025). In contrast, Tn*125* relies on IS*Aba125* and other classic transposon sequences to facilitate stable replication and transmission of *bla*_NDM-1_ (Wu et al., 2019). However, the lack of IS*Aba125* does not destabilize the spread of *bla*_NDM-1_; instead, IS*Kpn19* might provide an alternative mechanism for mobilization via replicative transposition or the “cut-and-paste” mechanism. This could potentially increases the recombination frequency of *bla*_NDM-1_ between plasmids but may also promote the evolution and spread of resistance genes through multiple pathways (Wu et al., 2016; Zhao et al., 2021).

In comparison, the *bla*_CTX-M-77_ gene is located on the chromosome, surrounded by IS*3* and IS*1A* insertion sequences. Chromosomal integration provides a stable inheritance mechanism and reduces the adaptive cost associated with plasmid maintenance. While *bla*_NDM-1_ on plasmids requires selective pressure to be maintained, it can spread rapidly. These findings reveal a dual evolutionary strategy in *K. ascorbata*: acquiring *bla*_NDM-1_ through plasmid-mediated transfer allows for rapid environmental adaptation, while chromosomal integration of *bla*_CTX-M-77_ ensures long-term stability. This complementary arrangement of resistance genes enables the bacteria to quickly respond to changes in antibiotic pressure and ensures the persistence of resistance traits (Zhao et al., 2021; Partridge et al., 2018; Oliveira et al., 2024; Liu et al., 2020; San Millan and MacLean, 2017).

From a clinical perspective, this dual strategy is concerning. The co-existence of plasmid-borne *bla*_NDM-1_ and chromosomal *bla*_CTX-M-77_ weakens the effectiveness of carbapenems and broad-spectrum cephalosporins, leaving limited treatment options, with polymyxin and tigecycline being among the few remaining choices. Furthermore, the presence of the novel IncR-IncFIA plasmid carrying *bla*_NDM-1_ may facilitate the rapid spread of carbapenem resistance, especially in hospital environments with high antibiotic pressure and patient-to-patient transmission risk, such as intensive care units. In our study, we observed a conjugative transfer efficiency of 3.87 × 10^−4^, confirming that this plasmid can transfer *bla*_NDM-1_ to recipient strains, further supporting its potential for rapid transmission in clinical settings (Wu et al., 2019; Kim et al., 2025; Yengui et al., 2025).

Genomic analysis of the L4110hy strain identified several virulence-related genes, including *nlpI*, *clpK1*, and *terC*. Among them, *nlpI* encodes the NlpI protein, an outer membrane lipoprotein that plays a role in cell wall stability and peptidoglycan degradation; its presence may reduce the efficacy of antibacterial agents by enhancing cell wall robustness (Wilson and Bernstein, 2016). *clpK1* belongs to the heat shock protein family and functions as a molecular chaperone, helping bacteria maintain protein homeostasis under high-temperature and host-induced stress conditions, thereby enhancing bacterial survival (Gurung and Biswas, 2022). *terC* is associated with tellurite resistance and confers the ability to withstand oxidative stress and metal toxicity (Whelan et al., 1995)^.^ These genes are associated with stress tolerance, protein homeostasis, and heavy metal resistance, possibly indirectly supporting the persistence of bacteria in harsh environments. The distribution of virulence factors, particularly on plasmids, suggests functional diversification, which may enhance the bacteria’s environmental adaptability and pathogenic potential. The presence of these virulence factors highlights the multifactorial nature of bacterial survival and resistance.

This study provides the first evidence of *bla*_NDM-1_ in *K. ascorbata*, expanding the host range of this gene and highlighting the need for monitoring non-traditional pathogens, particularly in high-selective-pressure environments like hospitals, to prevent the spread of resistance genes. Future research should focus on the transmission mechanisms of *bla*_NDM-1_, particularly the role of small mobile elements (such as transposons and insertion sequences) in this process, and further investigate their synergistic effects with other resistance genes. Studying the IS*Kpn19* insertion sequence and its role in resistance gene transfer will help unveil the evolutionary dynamics of resistance genes and their impact on public health. In conclusion, this study reveals the transmission mechanism of *bla*_NDM-1_ in *K. ascorbata* and provides theoretical support for future antibiotic resistance monitoring and control strategies.

## Data Availability

The datasets presented in this study can be found in online repositories. The names of the repository/repositories and accession number(s) can be found: https://www.ncbi.nlm.nih.gov/genbank/, the BioSample accession number of: SAMN44237076.

## Footnotes

^1^ https://www.eucast.org/

^2^ https://github.com/rrwick/Unicycler

^3^ https://rast.nmpdr.org

^4^ http://www-is.biotoul.fr/

^5^ https://cge.food.dtu.dk/services/ResFinder/

^6^ https://cge.food.dtu.dk/services/PlasmidFinder/

^7^ https://bioinfo-mml.sjtu.edu.cn/oriTfinder

^8^ http://www.mgc.ac.cn/VFs

^9^ https://blast.ncbi.nlm.nih.gov/Blast.cgi
